# Nephroprotective effect of vitamin D Against Levofloxacin-induced renal injury: an observational study

**DOI:** 10.25122/jml-2023-0096

**Published:** 2023-07

**Authors:** Abbas Muslim Mhaibes, Farah Kais Abdul-Wahab

**Affiliations:** 1 Department of Pharmacology and Toxicology, College of Pharmacy, University of Baghdad, Baghdad, Iraq

**Keywords:** Levofloxacin, oxidative stress, malondialdehyde, glutathione reductase, superoxide dismutase, renal damage, rats, vitamin D3, LFX: Levofloxacin, ROSs: Reactive oxygen species, OS: Oxidative stress, ELISA: Enzyme-linked immunosorbent assay, MDA: Malondialdehyde, HRP: Horse peroxidase, TMB: Tri-methyl benzidine, OD: Optical density, GSH: Glutathione, GR: Glutathione reductase, P: p-value

## Abstract

The pathogenesis of kidney damage involves complicated interactions between vascular endothelial and tubular cell destruction. Evidence has shown that vitamin D may have anti-inflammatory effects in several models of kidney damage. In this study, we evaluated the effects of synthetic vitamin D on levofloxacin-induced renal injury in rats. Forty-two white Albino rats were divided into six groups, with each group comprising seven rats. Group I served as the control (negative control) and received intraperitoneal injections of normal saline (0.5 ml) once daily for twenty-one days. Group II and Group III were treated with a single intraperitoneal dose of Levofloxacin (50 mg/kg/day) and (100 mg/kg/day), respectively, for 14 days (positive control groups). Group IV served as an additional negative control and received oral administration of vitamin D3 (500 IU/rat/day) for twenty-one days. In Group V, rats were orally administered vitamin D3 (500 IU/rat/day) for twenty-one days, and intraperitoneal injections of Levofloxacin (50 mg/kg/day) were administered on day 8 for 14 days. Group VI received oral vitamin D3 supplementation (500 IU/rat/day) for twenty-one days, followed by intraperitoneal injections of Levofloxacin (100 mg/kg/day) on day 8 for fourteen days. Blood samples were collected to measure creatinine, urea, malondialdehyde, glutathione reductase, and superoxide dismutase levels. Compared to the positive control group, vitamin D supplementation lowered creatinine, urea, and malondialdehyde levels, while increasing glutathione reductase and superoxide dismutase levels. Urea, creatinine, and malondialdehyde levels were significantly (p<0.05) higher in rats administered LFX 50mg and 100mg compared to rats given (LFX + vitamin D). The main findings of this study show that vitamin D reduces renal dysfunction, suggesting that vitamin D has antioxidant properties and may be used to prevent renal injury.

## INTRODUCTION

Levofloxacin (LFX) is a member of the class of antibiotics known as fluoroquinolones [1]. It has broad-spectrum bactericidal activity against Gram-positive and Gram-negative bacteria [2]. Adult infections, including sinus infections, bronchitis, pneumonia, and genitourinary infections, including both complex and uncomplicated urinary tract infections, are treated with Levofloxacin, the primary targets of which are topoisomerase IV and DNA gyrase, which impede bacterial cell division [3]. In addition, the fluoroquinolone class of antibiotics has been found to produce reactive oxygen species (ROSs), which may result in oxidative stress (OS), cellular damage to the kidney, and increased urea and creatinine [4, 5].

Vitamin D is a fat-soluble vitamin that comes in two primary forms: vitamin D2 (ergocalciferol), a product of plants, and vitamin D3 (cholecalciferol), a substance produced by the human body [6]. Vitamin D has DNA protective and antioxidant effects and modulates serum calcium homeostasis, immunological responses, antioxidant characteristics, and other physiological processes [7, 8]. Vitamin D controls various physiological processes, including kidney protection [9]. It exerts its effects by inhibiting fibrosis, inflammation, and apoptosis through the modulation of several pathways involved in kidney injury, such as the renin-angiotensin-aldosterone system (RAAS) [10], nuclear factor-κB (NF-κB), and transforming growth factor-β (TGF-β)/Smad. In addition to maintaining calcium and phosphate homeostasis [11], vitamin D is an essential modulator of cellular proliferation, inflammation, differentiation, and immune responses [12]. Furthermore, vitamin D insufficiency or deficiency can accelerate renal failure progression [13]. Studies have shown that vitamin D supplementation can prevent renal ischemia/reperfusion-induced kidney damage [14]. Moreover, vitamin D insufficiency has been associated with several diseases, including Alzheimer’s disease, Parkinson’s disease, multiple sclerosis, hypertension, and cardiovascular conditions [15]. These diseases are age-related and manifest later in life [16].

Reactive oxygen species (ROS) generation may be boosted by changes in mitochondrial energy metabolism that occur during vitamin D insufficiency, along with decreased antioxidant defenses. This combination may be a primary cause of age-related disorders worldwide [17]. Furthermore, vitamin D insufficiency can lead to imbalances in calcium metabolism and disruptions in redox cell signaling pathways. Given that adequate vitamin D levels may help maintain the usual decline in aging processes, it is plausible that vitamin D plays a crucial role in regulating the rate of aging [18]. One study examining the connection between vitamin D and calcium balance and oxidative stress concluded that calcium balance and mitochondrial membrane potentials were altered under oxidative stress. This alteration damages the mitochondria and DNA, causing the cell to undergo apoptosis or planned cell death. Apoptosis can be brought on by oxidative stress, mitochondrial abnormalities, disturbed intracellular electron balance, and inadequate antioxidant defenses [19]. The purpose of this research was to determine the impact of vitamin D3 on the oxidative stress markers and biochemical parameters present in the kidneys of rats.

## MATERIAL AND METHODS

### Sample size

The sample size for the study was determined using the computer application G*Power 3.1.9.7 (RRID: SCR 013726). With an effect size of 0.5, 95% power at a two-tailed alpha of 0.05, and a 95% confidence interval, the minimum total sample size was 42 rats (f). The formula used to calculate the sample size in this study is based on a comparison between groups when the endpoint is quantitative data.

Sample size = 2 SD2 (1.96 + 0.842) 2/d2

Where standard deviation (SD) = from previous studies or pilot study, d = effect size = difference between mean values [20].

### Study design and setting

The study was designed as an observational case-control study conducted from October 3, 2022, to January 8, 2022, using a convenient sample of rats. After two weeks of adaptation, the rats were randomly divided into six groups, with seven animals in each group, as follows:

1. Group I (negative control): Rats in this group received intraperitoneal injections (I.P.) of 0.5 ml of normal saline once daily for twenty-one days. This group served as the control group.
2. Group II (positive control): Rats were treated with a single dose of Levofloxacin (50 mg/kg/day) I.P. for 14 days [21].
3. Group III (positive control): Rats were treated with a single dose of Levofloxacin (100 mg/kg/day) I.P. for 14 days [21].
4. Group IV (negative control): Rats were orally administered a single dose of vitamin D3 (500 IU/rat/day) for twenty-one days [22].
5. Group V: Rats were orally administered vitamin D3 (500 IU/rat/day orally for twenty-one days, and I.P. levofloxacin (50 mg/kg/day) was injected on day 8 for 14 days [21, 22].
6. Group VI: Rats were treated orally with vitamin D3 500 IU/rat/day for twenty-one days, and I.P. levofloxacin 100 mg/kg/day injected at day 8 for fourteen days [21, 22].

### Materials

Levofloxacin and vitamin D3 were used in this study. Levofloxacin was acquired from Bader Pharm/Egypt with LOT number 210180, while vitamin D3 was acquired from ABIOGEN Pharma, Italy, with LOT number 02920, as shown in Table 1.

**Table 1:** Kits and medicines product summary

Providers	Kit	Catalog number	LOT number	Manufacturing data	Expiration date
Pars Biochem/China	MDA	PRS-30425Ra	202201	2022/1	2023/1
Pars Biochem/China	GR	PRS-30748Ra	202201	2022/1	2023/1
Pars Biochem/China	SOD	PRS-30597Ra	202201	2022/1	2023/1
RANDOX/UK	UREA	UR2316	506069	2022/10	2023/10
Biosystems/Spain	creatinine	11802	43065	2022/3	2024/3
Badr Pharma/Egypt	levofloxacin	28470/2020	210180	2022/1	2024/1
Abiogen/Italy	VitaminD3	036635011	02920	2020/4	2023/4
Euroclone S.P.A/ Italy	Phosphate buffered saline	ECB4004L	EUM009K	2022/6	2023/6

### Animals

A total of 42 white Albino rats of both sexes, weighing 150–250 grams and aged 10 weeks, were obtained from the Animal House of the Pharmacology and Toxicology Department, College of Pharmacy, University of Baghdad. The rats were housed in polycarbonate cages and placed in a room with a humidity of 60±5% and a temperature of 22±2 °C. The rats were provided commercial pellets and tap water, formulated to contain essential nutrients for a healthy and active lifestyle: balanced protein, fat, and carbohydrates, supplying the necessary energy for their daily functioning and performance. A 12/12-hour light-dark cycle was maintained throughout the experiment.

### Serum preparation and analysis

After slicing the carotid artery, blood was collected from the rats and placed in a gel tube. The tubes were allowed to stand undisturbed for 10 minutes before centrifugation for 20 min to collect the serum. After removing the top layer with a micropipette, the tube was refrigerated at -80 °C, and urea and creatinine tests were conducted [23].

### Determination of urea level

The urea concentration in the serum samples was determined using the enzymatic method based on the activity of the urease enzyme (Kaplan A). In this method, urea is broken down into ammonia and carbon dioxide in the presence of nitroprusside, sodium salicylate, and sodium hypochlorite. After the interaction between ammonia and sodium hypochlorite, chloramine is produced, which reacts with phenol to yield blue indophenols. The quantity of urea in the serum was measured in milligrams per deciliter (mg/dl), and the absorbance at 600 nm was used to monitor this process. The color remained constant for at least two hours [24].

### Determination of creatinine level

The creatinine in the sample reacted with picrate in alkaline media to generate a colorful complex (the Jaffe method). To prevent interference, the complex formation rate was quickly measured. It was determined that the proteins in serum and plasma samples react in an unspecific manner by analyzing the absorbance at 500 nm and measuring the serum creatinine levels in milligrams per deciliter (mg/dl) [25].

### Preparation of kidney tissue homogenate

After euthanizing rats with an anesthetic and then by cervical dislocation, a portion of the kidney was quickly resected, rinsed with phosphate-buffered saline to remove any clots or red blood cells, homogenized with phosphate buffer solution (1:9 w/v) (PH7.4) on ice, centrifuged at 4 °C and 3000 rpm for 20 min, and the supernatant was withdrawn and sent to analyze oxidative stress parameters (MDA, SOD, and GR) [26].

### Determination of malondialdehyde (MDA) content within the kidney tissue

To determine malondialdehyde (MDA) content within the kidney tissue, an enzyme-linked immunosorbent assay (ELISA) method was employed using ELISA kits and antibody sandwich techniques from PARS BIOCHEM. Firstly, purified rat malondialdehyde (MDA) antibodies were used to coat microtiter plate wells, creating solid-phase antibodies. The MDA samples were then added to the wells. To build the antibody-antigen-enzyme-antibody complex, the MDA antibody was combined with horse peroxidase (HRP)-labeled MDA antibody. The plate was incubated at 37°C for 30 minutes, followed by thorough washing and adding a tri-methyl benzidine (TMB) substrate solution. When an HRP-catalyzed TMB substrate becomes blue, the reaction is terminated by adding a sulfuric acid solution as a stop solution. The quantity of MDA in the samples was determined by comparing the optical density (OD) of the samples to the standard curve directly using ELISA, represented as mmol/L. The color shift from blue to yellow was quantified spectrophotometrically at a wavelength of 450 nm [27].

### Estimation of glutathione reductase (GR) enzyme within the kidney tissue

This test uses commercial assay kits from Pars Biochem and relies on sandwich methods for the ELISA reader. Coating microtiter plate wells with purified rat glutathione reductase (GR) antibody resulted in a solid-phase antibody. Then, GR and GR antibodies labeled with horseradish peroxidase were added to the wells (HRP). The antibody-antigen-enzyme-antibody complex was incubated for 30 min before washing. The reaction was triggered by adding TMB substrate, which becomes blue when the HRP enzyme is catalyzed in a dark environment. The response was subsequently terminated by adding sulfuric acid as a stop solution, and the color change was detected spectrophotometrically at 450 nm. The concentration of GR in the samples was determined by comparing the optical density (OD) of the pieces to a reference curve explicitly stated in nanograms per liter (ng/L) [28].

### Determination of super oxidase dismutase (SOD) enzyme within kidney tissue

This procedure was performed using sandwich ELISA. The rat SOD antibody supernatant was used to coat microtiter plate wells, creating solid-phase antibodies. The SOD samples were then added to the wells. To build an antibody-antigen enzyme-antibody complex, the SOD antibody was combined with HRP and then incubated at 37 °C for 60 min. The reaction was stopped by adding a sulfuric acid solution, causing the color of the substance to shift from red to yellow. The concentration of SOD in the samples was calculated by comparing the optical density (OD) of the samples to a reference curve at a wavelength of 450 nm. The SOD concentration was expressed in nanograms per liter (ng/L) [29].

### Histopathological studies

In this phase, the H&E (hematoxylin and eosin) staining technique was applied, and organs previously fixed in Bouine's fixative were dehydrated using 100 percent alcohol concentration. Subsequently, the organs were washed with xylene. The organs were sliced to a thickness of 5 mm using a rotary microtome and coated with paraffin wax. The samples were then stained with eosin-hematoxylin and photographed with a light microscope [30].

### Statistical analysis

Data were analyzed using the SPSS version 25 statistical program for Windows (RRID: SCR 016479). Descriptive statistics (means, standard deviations, frequencies, and percentages) were calculated for all study parameters. Kruskal Wallis test and pairwise comparisons were used to measure the differences in parameters that were not normally distributed across the study groups. One-way analysis of variance (ANOVA) and post hoc tests were used to measure the difference in the normally distributed parameters across the six study groups. The post hoc test used was Tukey's method. GraphPad Prism version 7.04 was used to develop figures (RRID: SCR_002798), and p-values<0.05 were considered statistically significant.

## RESULTS

### Effects on serum urea

There were significant differences in serum urea levels observed between the experimental groups. Rats in Group II had a statistically significant increase in serum urea levels (51.57±3.65 mg/dl) compared to Group I (42.43±3.21 mg/dl) (p<0.05). Similarly, Group III had significantly higher urea serum levels (58.00±2.77 mg/dl) compared to the control rats in Group I (42.43±3.21 mg/dl) (Table 2).

**Table 2:** Differences in urea levels across the study groups

Group no.	GPII (p-value)	GPIII (p-value)	GPIV (p-value)	GPV (p-value)	GPVI (p-value)
**GPI**	.004*	.000*	.831	.954	.005*
**GPII**		.040*	.001*	.058	.980
**GPIII**	.040*		.000*	.000*	.002*
**GPIV**	.001*	.000*		.253	.002*
**GPV**	.058	.000*	.253		.093

* Significant (p-value<0.05) according to ANOVA

### Effects on serum creatinine

**Figure 1:**
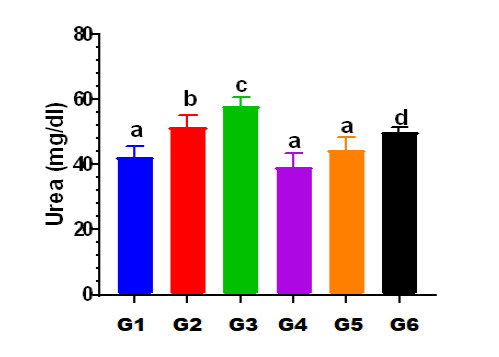
Differences in urea levels across the study groups G: Group values expressed in identical small letters (a) are not significantly different (p>0.05). Values expressed in non-identical small letters (a, b) are significantly different (p<0.05).

**Figure 2:**
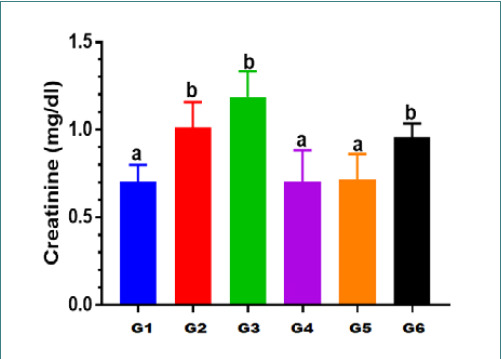
Differences in creatinine levels across the study groups G: Group values expressed in identical small letters (a) are not significantly different (p>0.05). Values expressed in non-identical small letters (a, b) are significantly different (p<0.05).

The creatinine level in Group II showed a statistically significant (p<0.05) increase in serum creatinine levels (1.01±0.15 mg/dl) compared to Group I (0.70±0.10 mg/dl) (Table 3). Furthermore, Group III had a statistically significant (p<0.05) increase in serum creatinine levels compared to Group I. However, in Group VI, rats treated with vitamin D3 (500 IU) and Levofloxacin (100 mg/kg/day) showed no significant reduction (p<0.05) in serum creatinine levels (0.9571±.07868 mg/dl) compared to the rats treated with Levofloxacin 100 mg alone (Group III) (1.19±0.15 mg/dl) (Table 4). In addition, rats in group V demonstrated a significant reduction (0.71±0.15 mg/dl) compared to group VI (0.95±0.07 mg/dl), as shown in Figures 1 and 2.

**Table 3:** Differences in creatinine levels across the study groups

Group no.	GPII (p-value)	GPIII (p-value)	GPIV (p-value)	GPV (p-value)	GPVI (p-value)
**GPI**	.010*	.000*	1.000	1.000	.003*
**GPII**		.413	.055	.032*	.997
**GPIII**	.413		.002*	.001*	.059
**GPIV**	.055	.002*		1.000	.092
**GPV**	.032*	.001*	1.000		.042*

* Significant (p-value<0.05) according to ANOVA

**Table 4:** Effects of Levofloxacin and vitamin D3 on serum renal function parameters

Group	Urea (mg/dl)	Creatinine (mg/dl)
**Group I**	42.43±3.21mg/dl a	0.70±0.10mg/dl a
**Group II**	51.57±3.65mg/dl b	1.01±0.15mg/dl b
**Group III**	58.00±2.77mg/dl c	1.19±0.15mg/dl b
**Group IV**	39.57±3.74mg/dl a	0.70±0.18mg/dl a
**Group V**	44.71±3.77mg/dl a	0.71±0.15mg/dl a
**Group VI**	50.00±1.528mg/dl d	0.9571±.07868mg/dl b

Each value represents Mean± standard deviation (M±SD); values expressed in non-identical small letters (a, b, c, and d) are significantly different (p<0.05)

### Effects on oxidative stress marker

#### Effect on malonaldehyde (MDA)

**Figure 3:**
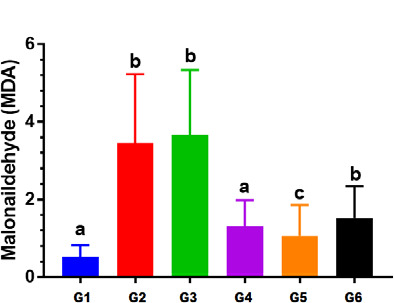
The difference in malonaldehyde (MDA) levels across the study groups G: Group values expressed in identical small letters (a) are not significantly different (p>0.05). Values expressed in non-identical small letters (a, b) are significantly different (p<0.05).

Animals treated with Levofloxacin (50 mg) once a day for 14 days (Group II) showed a statistically significant increase (3.4581±2.426 mmol/l) in MDA content compared to Group I (0.5264±3.342 mmol/l) (p<0.05), as shown in Table 5. In addition, rats treated with Levofloxacin (100 mg) once daily for 14 days (Group III) had a significant increase (p<0.05) in MDA content (3.6732±4.287 mmol/l) compared to Group I (0.5264±3.342 mmol/l) (Figure 3). Although there were no significant (p>0.05) differences in MDA contents in renal tissue homogenate between Group IV and Group I, rats treated with a single dose (500IU) of vitamin D3 and Levofloxacin (50 mg/kg) once a day (Group V) had a significant reduction in MDA levels (p<0.05) compared to rats treated with Levofloxacin (50 mg) alone (Group II).

**Table 5:** Effects of vitamin D3 and Levofloxacin on oxidative stress markers

Group	MDA Median ± Interquartile rang	GR mean ±SD	SOD mean ±SD
**Group I**	0.52±3.3 a	47.36±15.11 a	50.22±23.02 a
**Group II**	3.45±2.4 b	14.96±6.93 b	17.15±5.20 a
**Group III**	3.67±4.2 b	10.22±3.65 b	14.78±5.23 a
**Group IV**	1.31 ± 3.5 a	41.1±9.993488 a	37.3±12.2 a
**Group V**	1.06 ± 1.4 c	36.5±9.894181 c	42.7±18.1 a
**Group VI**	1.51 ± 2.3 b	30.1±9.186323 d	39.03±9.9 b

Values are expressed in identical small letters: (a) non-significant difference. However, values expressed in non-identical small letters (a, b) are significantly different (p<0.05).

#### Effect on Glutathione reductase (GR)

**Figure 4:**
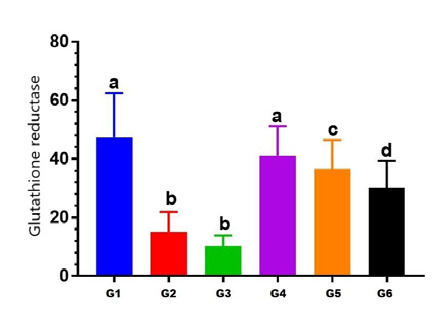
The difference in the glutathione reductase (GR) levels across the study groups G: Group values expressed in identical small letters (a) are not significantly different (p>0.05). Values expressed in non-identical small letters (a, b) are significantly different (p<0.05).

The level of glutathione enzyme in animals treated with Levofloxacin (50 mg/kg/day) (Group II) showed a statistically significant reduction (p<0.05) in GR enzyme levels in rat renal tissue homogenate compared to the corresponding enzyme activity level in control (Group I) rats, as shown in Table 5. Rats treated with Levofloxacin (100 mg/kg/day) (Group III) showed a significant reduction (p<0.05) in GR enzyme levels compared to Group I (Figure 4).

#### Effect on superoxide dismutase (SOD)

**Figure 5:**
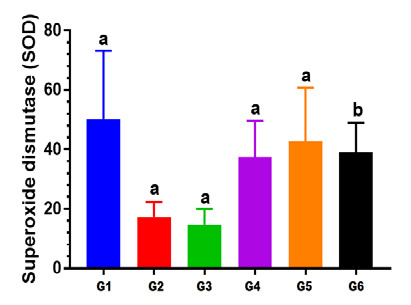
The difference in the superoxide dismutase (SOD) levels across the study groups G: Group values expressed in identical small letters (a) are not significantly different (p>0.05). Values expressed in non-identical small letters (a, b) are significantly different (p<0.05).

The study showed that the level of SOD in Group II showed a statistically non-significant reduction (p<0.05) in the rat tissue kidney homogenate compared to Group I, as shown in Table 5. Furthermore, rats in Group III showed a non-significant reduction (p<0.05) in SOD enzyme levels compared to Group I (Table 5 and Figure 5).

### Histopathology

**Figure 6:**
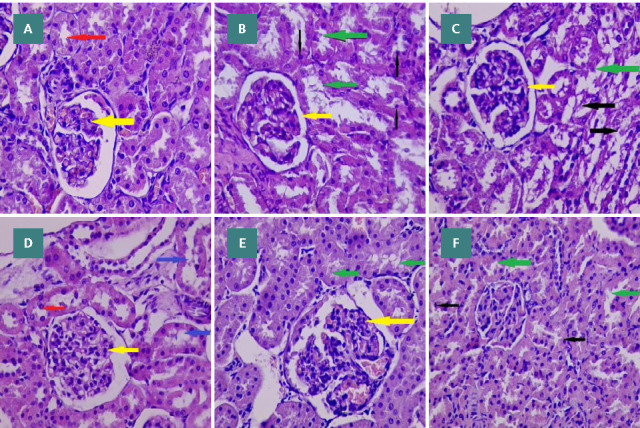
Effect of levofloxacin and vitamin D3 on kidney histopathology (A) Control group with typical histological structure of tubules; (B) Group II showed degenerative changes with apoptotic cells of renal tubules; (C) Group III showed that the majority of tubules had degenerative changes and damage to epithelial cells; (D) Group IV showed a typical histological structure of renal tubules; (E) Group V showed renal tubules with mild degenerative changes; and (F) Group VI showed very mild degenerative changes in the epithelial lining of renal tubules with rare apoptotic cells. The yellow arrow marks (glomeruli), the blue arrow marks distal convoluted tubules, the red arrow marks proximal convoluted tubules, the green arrow marks apoptotic cells, and the black arrow marks degenerative changes.

Histopathological analysis revealed distinct histological differences among the experimental groups. Group I (control) exhibited typical histological structures of renal tubules. In Group II, degenerative changes were observed, characterized by apoptotic cells in the lining epithelium of renal tubules. In Group III, the majority of tubules showed degenerative changes and damage of epithelial cells (i.e., no more lining epithelial cells seen). Group IV displayed histological structures like the typical renal tubules. Group V showed mild degenerative changes within renal tubules. Finally, Group VI showed minimal degenerative changes in the epithelial lining of renal tubules, with rare apoptotic cells (Figure 6 A-F).

## DISCUSSION

The kidney is the primary excretory organ in humans and eliminates neurotoxic xenobiotic and metabolic waste from the body [31]. In addition, the kidney regulates blood pressure (BP) and blood volume by preserving electrolytes, secreting certain hormones (such as erythropoietin and renin), and maintaining acid-base balance [32]. Reactive oxygen species produced by fluoroquinolone antibiotics have been linked to oxidative stress, cellular damage, and kidney damage [33]. In this study, the administration of Levofloxacin at two different doses (50 mg/kg/day and 100 mg/kg/day) induced considerable kidney damage, oxidative stress, and depletion of renal antioxidant reserves. The glomeruli of the kidneys easily filter both urea and creatinine, which are metabolic waste products. Monitoring the serum concentrations of these substances is commonly used as a diagnostic tool to assess renal function and detect potential renal disorders [34, 35]. It has been noted that plasma urea rises in both acute and chronic intrinsic renal dysfunction. When the effective circulating blood volume is lowered along with renal perfusion [36], elevated creatinine and urea plasma levels are signs of impaired renal function [37]. Numerous defense systems exist in cells to prevent the harmful effects of ROS, including enzymes that scavenge free radicals and stop chain reactions, such as glutathione reductase (GR), vitamins C and E, SOD, CAT, and GSH peroxidase [38]. A buildup of superoxide ions and hydrogen peroxide caused by inhibiting these defense mechanisms or a decrease in their activity would make cells more susceptible to cellular damage caused by free radicals [39]. Superoxide ions (O2) are removed by SOD by converting them into hydrogen peroxide (H2O2), in which GSH can be quickly converted to water and oxygen [40]. Under normal circumstances, SOD protects cells from O2- by having high catalytic activity and is present at high concentrations across all tissues [41]. The vulnerability of the cell to O2- attack may rise due to the decreased SOD and GR activity observed in this study. The disintegration of H2O2 produced by the reactions of SOD with oxygen and water is catalyzed by GR [42]. Therefore, when the activity of GR and SOD is reduced by the drug, it may increase the susceptibility of the kidneys to oxidative stress induced by H2O2 and hydroxyl radicals.

Vitamin D3 is a lipid-soluble molecule that scavenges physiologically relevant free radicals by increasing the levels of SOD and GR, which react with oxygen radicals [43]. The cell becomes more vulnerable to free radicals due to the decrease in SOD and GR activity brought on by the administration of LFX. The level of reduced glutathione reductase (GR) serves as an indicator of the cellular redox state. Previous studies have indicated a correlation between fluoroquinolones and the generation of oxidative stress, leading to decreased antioxidant capacity [44]. The increased MDA generation in this study suggests that the oxidative cell damage caused by free radicals plays a role in mediating fluoroquinolone toxicity. This finding is consistent with several research results [44, 45]. This research focuses on the preventive impact of vitamin D against Levofloxacin-induced kidney injury in rats by inhibiting oxidative stress by decreasing MDA levels and raising GSH and SOD levels.

The Levofloxacin-treated group (Groups II and III) had significantly higher levels of urea and creatinine than the control group (Group I) and the vitamin D-treated group (Groups IV, V, VI) (p<0.05). Investigators identified the primary route of Levofloxacin excretion through the kidneys [46]. In addition, previous studies demonstrated that the administration of higher concentrations of Levofloxacin results in higher drug concentrations in plasma and urine and that at an acute and extremely toxic dose, the elimination of Levofloxacin is interrupted, increasing serum creatinine concentration, which is one of the biomarkers of renal failure. The present research supports these findings [47,48]. Plasma levels of urea and creatinine were found to be significantly lower (p<0.05) in groups IV, V, and VI compared to groups II and III. These findings are consistent with the conclusions of Zhixia Song *et al*. [21], who reported that vitamin D supplementation improves creatinine clearance and renal blood flow, decreasing creatinine and urea levels [49]. The present research demonstrated a significant difference (p<0.05) in the concentration of MDA, GR, and SOD in kidney tissue homogenate between the control (group I), Levofloxacin-treated groups (II, III), and vitamin-D treated groups (IV, V, VI), which is consistent with prior animal studies. The administration of Levofloxacin resulted in a significant increase in MDA levels and a decrease in GR and SOD levels compared to the control group (Group I) and the vitamin D-treated groups (Groups IV, V, VI), which might be related to oxidative stress [28, 50]. These findings align with earlier research that has demonstrated the antioxidant function of vitamin D, which regulates the formation of free radicals by reactive species of nitric oxide and oxygen [51, 52].

## CONCLUSION

This research established the positive effects of vitamin D3 on levofloxacin-induced nephrotoxicity by improving oxidative markers (MDA), antioxidants (GR) (SOD) in kidney homogenates, compensating for the increase in urea and creatinine levels, and improving histological lesions in rat kidneys. These findings suggest a potential method for preventing levofloxacin kidney injury by giving vitamin D3.

## Data Availability

The data supporting the findings of this study are available at Zenodo with the following DOI: 10.5281/zenodo.7738717. The dataset, titled “Measurement of renal failure biomarkers for the Nephroprotective Effect of Vitamin D Against Levofloxacin-Induced Renal Injury: an observational study,” is accessible and can be downloaded. The data is provided under the Creative Commons Attribution 4.0 International license (CC-BY 4.0).

## Personal thanks

We would like to express our sincere gratitude to the laboratory team at Salem Rashid's laboratory for their valuable contributions to this study.
